# A Treatise, Practical and Theoretic, on Cancers and the Cancer Process

**Published:** 1894-06

**Authors:** 


*A Treatise, Practical and Theoretic, on Cancers and the Cancer Process.* By HERBERT SNOW, M.D. Pp. xv., 384. London: J. & A. CHURCHILL. 1893.

The first thing which strikes us in glancing over this treatise is the abundant reference to a few previous books and papers by the same author, who evidently desires that the reader should duly appreciate his labours.

On turning to the discussion of still doubtful points, and in particular to that of the parasitic theory of cancer, we find the following: "The reproduction of Lieberkühn's follicle structure in an aberrant form throughout the secondary metastases of gastric and intestinal cancer is one of the most obvious and overwhelming arguments which controvert the microbe-theory of cancer genesis. It is impossible to believe that an organism introduced into the body from without can possibly cause an elaborate tissue arrangement to be thus copied in distant parts" (p. 161). To the ordinary mind it would appear that the resemblances of metastases to the original growth is merely the result of heredity, and cannot be cited as evidence either for or against a parasitic theory. On page 9, regarding Ruffer's researches, Dr. Snow says: "There is no evidence in favour of the parasitic nature of the bodies therein described." This is rather "flat," but inconclusive.

Dr. Snow's own theory is that the cancer process consists essentially in a local cell-rebellion, certain cells casting off their allegiance to the central authority of the nervous system—a theory which certainly has the merit that no one can either prove or disprove it.

For the rest, the book first deals with the structural anatomy of particular species of cancer, and secondly with malignant disease in particular organs or parts, with the most fitting measures of cure or relief.

